# In Vivo Evaluation of the Wound Healing Activity of Extracts and Bioactive Constituents of the Marine Isopod *Ceratothoa oestroides*

**DOI:** 10.3390/md18040219

**Published:** 2020-04-18

**Authors:** Evgenia Sofrona, Leto-Aikaterini Tziveleka, Maria Harizani, Panagiota Koroli, Ioannis Sfiniadakis, Vassilios Roussis, Michail Rallis, Efstathia Ioannou

**Affiliations:** 1 Section of Pharmacognosy and Chemistry of Natural Products, Department of Pharmacy, School of Health Sciences, National and Kapodistrian University of Athens, 15771 Athens, Greece; evgenia.sofrona@gmail.com (E.S.); ltziveleka@pharm.uoa.gr (L.-A.T.); mariachariz@pharm.uoa.gr (M.H.); roussis@pharm.uoa.gr (V.R.); 2 Section of Pharmaceutical Technology, Department of Pharmacy, School of Health Sciences, National and Kapodistrian University of Athens, 15784 Athens, Greece; giotakor17@hotmail.com; 3 Athens Naval Hospital, Pathologoanatomic Laboratory, 11521 Athens, Greece; jsfiniadakis@yahoo.gr

**Keywords:** *Ceratothoa oestroides*, marine isopod, wound healing, SKH-hr1 hairless mice, eicosapentaenoic acid

## Abstract

Wound healing is a fundamental response to tissue injury and a number of natural products has been found to accelerate the healing process. Herein, we report the preparation of a series of different polarity (organic and aqueous) extracts of the marine isopod *Ceratothoa oestroides* and the in vivo evaluation of their wound healing activity after topical administration of ointments incorporating the various extracts on wounds inflicted on SKH-hr1 hairless mice. The most active extract was fractionated for enrichment in the bioactive constituents and the fractions were further evaluated for their wound healing activity, while their chemical profiles were analyzed. Wound healing was evaluated by clinical assessment, photo-documentation, histopathological analysis and measurement of biophysical skin parameters, such as transepidermal water loss (TEWL), hydration, elasticity, and skin thickness. The highest levels of activity were exerted by treatment of the wounds with a fraction rich in eicosapentaenoic acid (EPA), as well as myristic and palmitoleic acids. Topical application of the bioactive fraction on the wounds of mice resulted in complete wound closure with a skin of almost normal architecture without any inflammatory elements.

## 1. Introduction

A wound is a physical injury created through contusion, hematoma, lacerations or abrasions that leads to loss of the epithelial integrity of the skin [1]. Wound healing is a complex and dynamic process that involves various types of cells, including leukocytes, blood cells, fibroblasts, and epithelial cells, as well as mediators, growth factors, and cytokines [2,3]. Generally, the healing of a wound injury proceeds through four distinct but overlapping phases. It starts with hemostasis (24 h), followed by inflammation (2–5 days), continues with angiogenesis, extracellular matrix deposition, granulation tissue formation and epithelialization during the proliferative phase (5 days–2 weeks), and is completed with connective tissue deposition during the tissue remodeling/maturation phase (3 weeks–24 months) [1,4,5,6,7].

Most wounds heal naturally. Nevertheless, under some conditions wound healing is prolonged. This prolongation results in chronic wounds with more severe wound conditions and therefore, therapeutic treatments, involving bioactive healing agents and/or wound dressings, are employed to facilitate the healing process [8].

Nature is considered an endless source of bioactive molecules. Even today, natural products are still considered as ideal medicines for a wide range of diseases. Naturally derived bioactive molecules that exhibit an array of biological activities hold potential for medical, pharmaceutical, and biotechnological applications in wound healing and skin regeneration [9]. The effect of natural compounds derived from medicinal plants of terrestrial origin, such as essential oils, terpenoids, saponins, fatty acids, alkaloids, flavonoids, tannins, and phenols, on wound healing has been frequently evaluated and their mechanisms of action have been investigated in a number of studies [10].

In the recent years, marine organisms have been proven a treasure trove of chemodiversity and a rich reservoir of potentially useful substances with medicinal properties, such as antibacterial, antifungal, antiviral, anti-inflammatory, anticoagulant, and antineoplastic activity [11,12,13,14,15]. Nowadays, there is a constantly increasing number of studies targeting the discovery of compounds isolated from marine organisms effective in wound healing and skin tissue regeneration applications [16,17,18].

The marine isopod *Ceratothoa oestroides* is a parasite found in the buccal cavity of fish, causing serious lesions and often leading to the death of the host, especially of young fish [19,20]. Recently we have demonstrated the significant in vivo wound healing potential of a *C. oestroides* oily extract [21,22]. Herein, a series of different polarity (organic and aqueous) extracts of *C. oestroides* was prepared and evaluated for their wound healing activity on SKH-hr1 hairless mice, while the most active extract was fractionated for enrichment in the bioactive constituents. The fractions were further evaluated for their wound healing activity and their chemical profiles were analyzed. Clinical evaluation, photo-documentation and histopathological analyses of the skin were performed and parameters, such as hydration, transepidermal water loss (TEWL), elasticity, and skin thickness, were assessed.

## 2. Results and Discussion

### 2.1. Preparation of Extracts and Evaluation of their Wound Healing Activity

Specimens of the marine isopod *C. oestroides* were exhaustively extracted with mixtures of CH_2_Cl_2_/MeOH to afford after evaporation of the solvents in vacuo an orange oily residue (total organic extract, COTOE) that was sequentially partitioned in water and organic solvents of increasing polarity (cHex, CH_2_Cl_2_, and *n*-BuOH) to afford four extracts of different polarity, namely cHex (COA), CH_2_Cl_2_ (COB), *n*-BuOH (COC), and H_2_O (COD) extracts.

Wounds were induced on SKH-hr1 hairless male mice by surgically removing a 1 cm^2^ piece of skin from the dorsal region of each mouse and were treated daily by topical application of ointments incorporating 1% of the five above-mentioned extracts, as well as an ointment containing 1% *C. oestroides* olive oil extract (COOE) previously investigated [21] for comparison purposes. Additionally, a group of mice receiving no topical treatment was used as negative control. The wound healing progress was evaluated primarily on the basis of daily clinical observation, as well as photo-documentation and histopathological assessment at the end of the experiment, while measurements of hydration, TEWL, elasticity and skin thickness were also taken into account.

**Figure 1:**
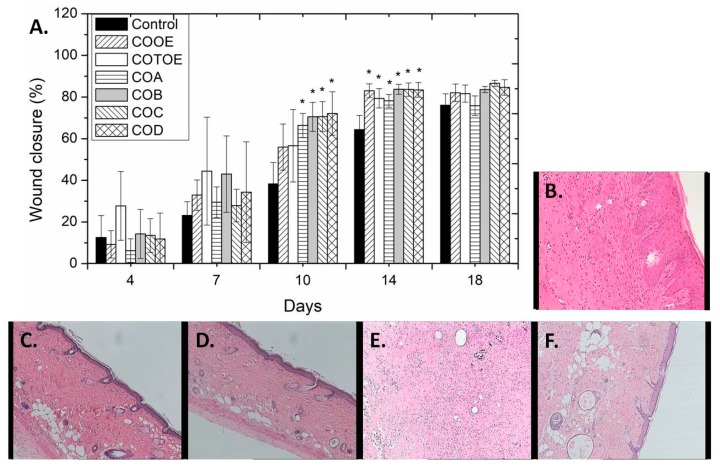
(**A**) Time course of the in vivo wound healing effect of different treatments (no treatment, control; 1% olive oil extract, COOE; 1% total organic extract, COTOE; 1% cHex extract, COA; 1% CH_2_Cl_2_ extract, COB; 1% *n*-BuOH extract, COC; 1% H_2_O extract, COD) on mice, expressed as % of wound closure and evaluated for 18 days. Values are presented as the mean ± SD (n=3–4 mice per group). One-way ANOVA followed by Tukey’s test was applied for comparison between the extract-treated mice groups and the negative control (* *p* < 0.05 vs control). (**B**–**F**) Representative histopathological images of mice skin on day 18 without treatment (**B**) and after treatment with ointments containing 1% COTOE (**C**), 1% COB (**D**), 1% COC (**E**) and 1% COD (**F**) extracts (magnification 100× (**A**, **C**, **D** and **F**) and 200× (**B** and **E**)). Samples were stained with hematoxylin and eosin.

From the 7^th^ day after wound induction and onwards, the healing activity of all *C. oestroides* extracts was greater than that observed for the negative control group. On the 10^th^ day, the healing activity of the ointments containing COB, COC, and COD extracts was significantly better as compared to that of the negative control group, whereas on the 14^th^ day significant healing was exerted by all extracts tested (Figure 1A). Taking into account that one day in mice life corresponds to 1–2 months in humans, the acceleration of wound healing observed up to day 14 (in comparison to the negative control group) can be translated to a significant positive effect of the extracts on the reduction of the time needed to achieve wound closure. After a time point, physiological healing takes place anyway (unless other co-morbidities exist) and differences are expected to cease being statistically significant. Additionally, TEWL measurements at the end of the experiment (day 18) were conducted in order to examine the epidermal barrier integrity of the differently treated groups. The measurements revealed small differences in the mean TEWL values for all groups during the healing process as compared to the negative control group, indicating relatively improved epidermal barrier integrity for the groups treated with *C. oestroides* extracts (Supplementary Figure S1).

Even though the percentage of wound closure is indicative of the wound healing process, histopathological observations of representative skin biopsies provide necessary and reliable information for the extent of inflammation in the area of the wound. Therefore, at the end of the experiment, mice from all groups were sacrificed, skin tissue was excised and skin sections were analyzed for the observation of important parameters, such as inflammation, hyperkeratosis, parakeratosis and skin structure. Skin sections of the different treatment groups are depicted in Figure 1B–F. The negative control group showed moderate lymphocytic infiltrations; in contrast, very few inflammatory elements were detected for the groups treated with 1% COB and 1% COD extracts, with the latter however showing also the presence of scattered lymphocytes. In the case of the group treated with 1% COOE extract, moderate inflammation with mild fibroblastic reaction could be detected. On the contrary, treatment with 1% COA, 1% COC, and 1% COTOE extracts exerted intense inflammation on the respective mice groups. More specifically, intense inflammation with fibroblastic reaction was detected in the group treated with COA extract, moderately intense lymphocytic infiltrations associated with foreign body type giant-cell granulomata, sparse neutrophils and macrophages and microcalcifications were observed for the group treated with COC extract, whereas in the group treated with COTOE extract obvious was the presence of inflammatory crust and inflammatory elements throughout the skin, as well as the induction of fibroblastic reaction in the dermis. On the basis of these results, COB, COD, and COOE extracts were considered promising and were selected for further analysis.

**Figure 2:**
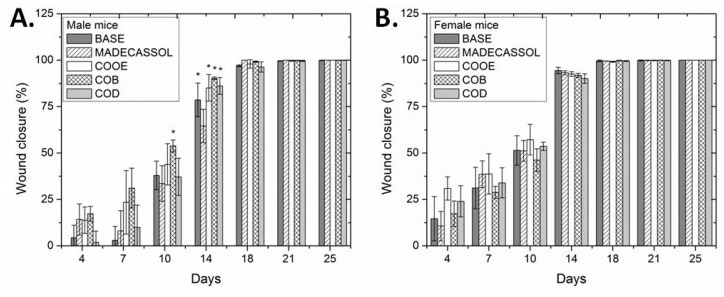
The effect of the different treatments (ointment base, BASE; Madecassol; 1% olive oil extract, COOE; 1% CH_2_Cl_2_ extract, COB; 1% H_2_O extract, COD) on the wound healing process on male (**A**) and female (**B**) mice, expressed as % of wound closure and evaluated for 25 days. Values are presented as the mean ± SD (n=7 mice per group). One-way ANOVA followed by Tukey’s test was applied for comparison between the extract-treated mice groups and the negative control (* *p* < 0.05 vs control).

In order to further investigate the wound healing potential of ointments containing 1% COB, COD and COOE extracts, a further experiment was conducted using both male and female mice for longer periods (up to 25 days). Additionally, a group treated with the ointment base without the incorporation of any extract (BASE) and a group treated with a commercially available agent (Madecassol cream) were included in the experiment for comparison reasons. It was seen that although the wounds healed faster in female mice (14^th^ day), statistically significant differences were observed only in male mice. Specifically, on the 10^th^ day of the wound healing process, COB extract exerted significantly better activity on male mice than the positive control, Madecassol, while on the 14^th^ day all groups treated with *C. oestroides* extracts showed improved wound healing in comparison to that treated with Madecassol (Figure 2A). On the contrary, in female mice no such differences were observed (Figure 2B). All other examined parameters (TEWL, hydration, elasticity and skin thickness) did not show any significant differences between male and female mice (Supplementary Figure S2).

Complementary, observations of histopathological sections showed that for both the ointment base- and Madecassol-treated mice groups no inflammation was detected in female mice, while in male mice intense inflammation in deep dermis was observed for the ointment base-treated group and mild inflammatory infiltrations were evident for the Madecassol-treated group (Supplementary Figure S3). Diffused inflammatory infiltrations of limited extent but throughout the skin were observed in male mice, whereas mild inflammation, skin thickening to a limited extent and the presence of many keratohyalin granules were detected in female mice, when both groups were treated with COOE extract. In the case of COB- and COD-treated mice, marginal inflammation was observed for both male and female mice, albeit it could be seen that gender plays an important role in the healing process, with female mice healing much faster and easier than male. Therefore, male mice were selected for further evaluation of COB and COD extracts. Analysis of the ^1^H NMR spectra of COB and COD extracts revealed the presence of fatty acids and carbohydrates, respectively.

In a subsequent experiment, COD extract, incorporated in a gel formulation designed for aqueous extracts, was tested at different concentrations (1%, 2%, and 4%) and found to have similar activity to the one exerted by the gel base (data not shown) and was therefore excluded from further testing.

### 2.2. Fractionation of COB Extract and Evaluation of the Wound Healing Activity of the Obtained Fractions

Since COB extract was systematically proven to be more efficient in the healing of wounds of mice in comparison to all other *C. oestroides* extracts tested, it was subjected to chromatographic separations to afford three fractions (COB-A, COB-B, and COB-C) exhibiting different chemical profiles. Specifically, the ^1^H NMR spectra of the three fractions revealed the presence of fatty acids differing in the degree of unsaturation, as well as the presence of triglycerides only in the case of COA-C (Supplementary Figures S4–S6). Methylation and GC-MS analysis of the three fractions revealed that COB-A consisted mainly of eicosapentaenoic acid (EPA, 61.8%), myristic acid (14.7%) and palmitoleic acid (12.2%), COB-B contained mainly oleic acid (54.1%), palmitic acid (30.1%), and linoleic acid (15.8%), whereas COB-C contained mainly palmitic acid (14.1%) and higher fatty acids.

**Figure 3:**
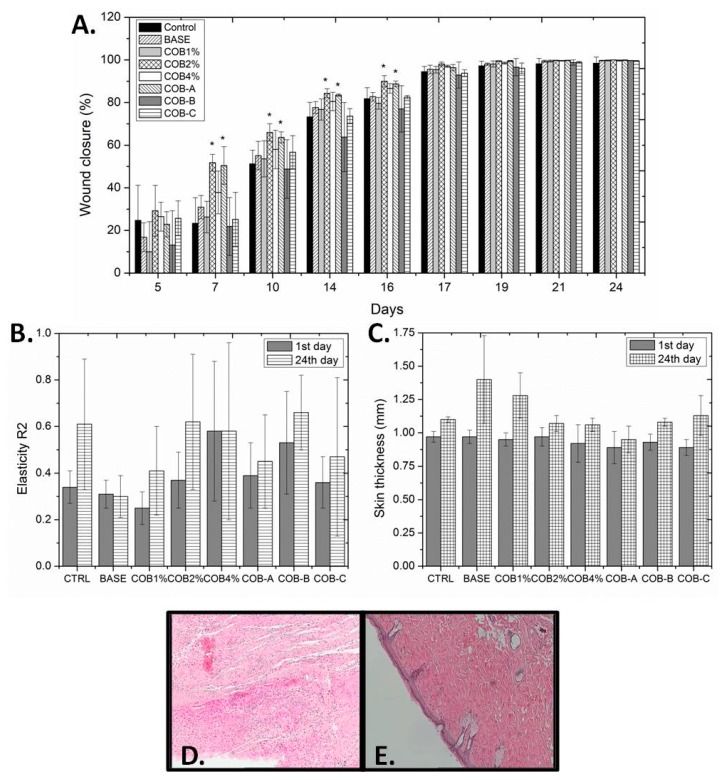
(**A**) Wound closure (%) as a function of the treatment duration in mice with different treatments (no treatment, control; ointment base, BASE; 1%, 2%, and 4% CH_2_Cl_2_ extract, COB1%; COB2% and COB4%, respectively; 0.3% COB-A fraction, COB-A; 0.3% COB-B fraction; COB-B; 0.3% COB-C fraction, COB-C). (**B**) Elasticity values for the various mice groups on day 1 and day 24 of the experiment. (**C**) Skin thickness values (mm) for the various mice groups on day 1 and day 24 of the experiment. Values are presented as the mean ± SD (n=4 mice per group). One-way ANOVA followed by Tukey’s test was applied for comparison between the treated mice and the control (* *p* < 0.05 *vs* control). (**D** and **E**) Representative histopathological images of mice skin on day 24 without treatment (**D**) and after treatment with ointment incorporating 0.3% COB-A fraction (**E**) (magnification 100× (**E**) and 200× (**D**)). Samples were stained with hematoxylin and eosin.

Subsequently, the three obtained fractions (at a concentration of 0.3%), along with COB extract incorporated at different concentrations (1%, 2%, and 4%), were evaluated on male mice. A statistically significant difference in the wound closure process was observed between the group treated with the ointment base and the groups treated with ointments containing 2% COB extract or 0.3% COB-A fraction (Figure 3A). Improved wound healing activity was observed between the 7^th^ to 16^th^ day of treatment. After this period, an almost complete healing (> 90%) was observed for all extracts and fractions that reached 100% on the 24^th^ day, except in the case of the negative control group.

As far as the TEWL is concerned, the best values were observed for the group on which 2% COB extract was applied, followed by that of 0.3% COB-A fraction (Supplementary Figure S7A). The hydration measurements showed no significant differences among the different formulations (Supplementary Figure S7B), while the elasticity of all examined groups was better at the end of the treatment (Figure 3B), with the exception of the groups treated with either the ointment base or 4% COB extract. Finally, the skin thickness of mice at the end of the experiment remained constant in most cases, except when either the ointment base or 1% COB extract were applied on the wound (Figure 3C).

Examination of histopathological sections for the various groups showed that mice treated with 2% COB extract showed a totally healthy epidermis, albeit the formation of an absorbent-type granuloma in the dermis and deposition of calcium salts and hemosiderin were evident. In contrast, the groups treated with 1% and 4% COB extract did not lead to significant reduction of inflammation and restoration of skin architecture. Furthermore, the group treated with COB-A fraction did not show any signs of inflammation, while at the same time stimulation of fibroblasts’ activity was observed (Figure 3D,E). For the groups treated with COB-B and COB-C fractions, moderate inflammation was observed.

**Figure 4:**
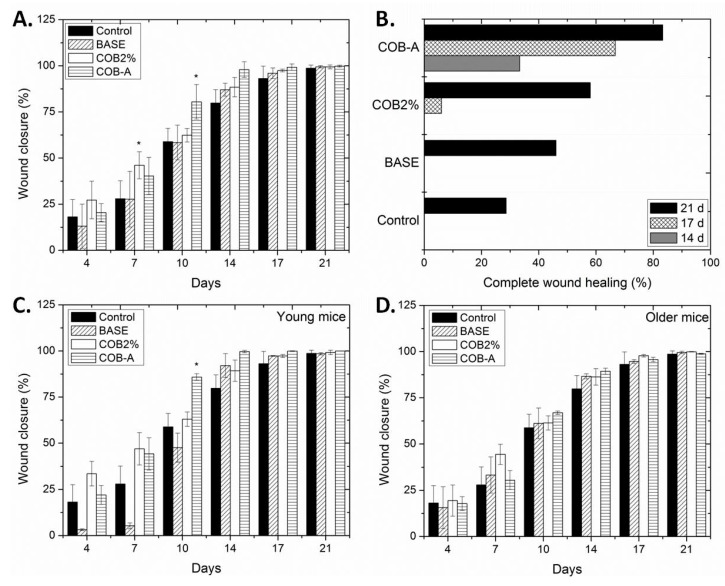
The effect of the different treatments (no treatment, control; ointment base, BASE; 2% CH_2_Cl_2_ extract, COB2%; 0.3% COB-A fraction, COB-A) on the wound healing process on young and older mice. Wound closure (%) is expressed as a function of the treatment duration in the mixed population (**A**), young (**C**), and older (**D**) mice. Values are presented as the mean ± SD. One-way ANOVA followed by Tukey’s test was applied for comparison between the extract-treated mice groups and the negative control (* *p* < 0.05 vs control). (**B**) The percentage of the complete wound healing of mice observed on day 14, day 17 and day 21 as a result of the different treatments.

**Figure 5:**
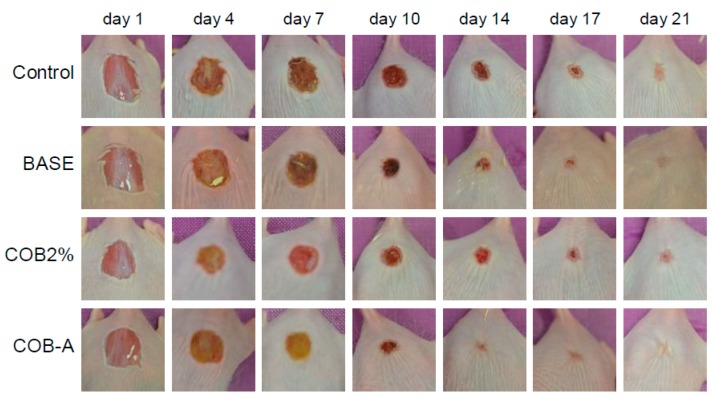
Representative images of the wound areas of the various mice groups (no treatment, control; ointment base, BASE; 2% CH_2_Cl_2_ extract, COB2%; 0.3% COB-A fraction, COB-A), as recorded over a 21-days period.

According to these results, the most promising ointment formulations incorporating 2% COB extract and 0.3% COB-A fraction, along with the ointment base were selected for further testing in a larger number of mice. Although the average age of mice was 2 months, two groups, one of young (1–2 months) and one of older (3–4 months) mice, could be distinguished. Such variation represents a 15 years difference in human age. Statistically significant differences in the wound closure values were detected among the different groups examined (Figure 4A). The percentage of the healed mice of the different groups was also denoted as a function of the days required for complete wound closure (Figure 4B). The observed differences depended on the age of the mice examined, with young mice showing enhanced and faster wound healing ability (Figure 4C,D). Overall, the best results were observed for the mice treated with the ointment formulation incorporating COB-A fraction. Even though slightly higher levels of wound closure were observed for the groups (both young and older) treated with the 2% COB extract for the first 7 days, the % of complete wound healing, as depicted in Figure 4B, is striking for the COB-A- treated mice, since even on day 14 more than 30% have healed completely. On the contrary, the wounds of mice treated with the 2% COB extract started to become completely healed only on day 17 (less than 10%). Representative images of the wound healing process in young mice are shown in Figure 5.

**Figure 6:**
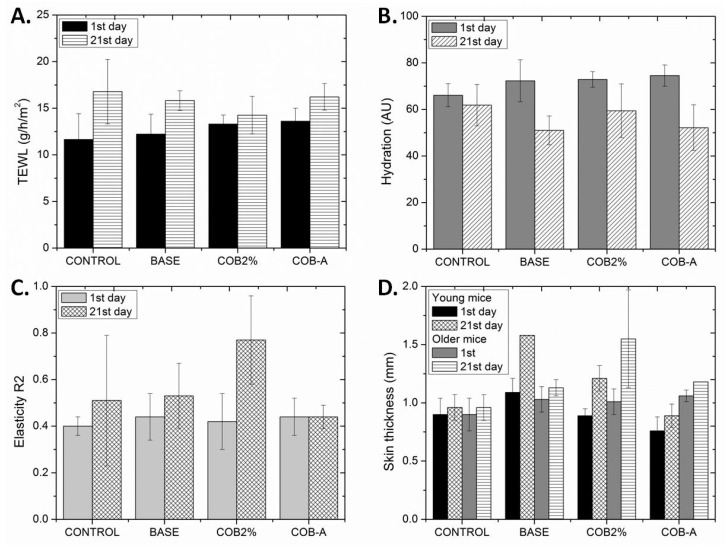
(**A**) Transepidermal water loss (TEWL) values for the various mice groups (no treatment, control; ointment base, BASE; 2% CH_2_Cl_2_ extract, COB2%; 0.3% COB-A fraction, COB-A) on day 1 and day 21 of the experiment. (**B**) Hydration values for the various mice groups on day 1 and day 21 of the experiment. (**C**) Elasticity values for the various mice groups on day 1 and day 21 of the experiment. (**D**) Skin thickness values (mm) for the various mice groups on day 1 and day 21 of the experiment. Values are presented as the mean ± SD (n = 9 mice per group). One-way ANOVA followed by Tukey’s test was applied for comparison between the treated mice and the control.

The TEWL and hydration measurements did not show any significant differences among the different formulations examined (Figure 6A,B). Nevertheless, it has been proven that the lack of polyunsaturated fatty acids (PUFAs) can cause increased TEWL, resulting in skin barrier function deficiency [23]. The elasticity of all examined groups was fairly better at the end of the experiment (Figure 6C), with the group treated with the 2% COB extract showing the best elasticity value. On the other hand, the skin thickness of mice at the end of the experiment remained almost constant relative to the start of the experiment in the negative control and the COB-A- treated groups, while in the case of either the ointment base- or 2% COB-treated groups a significant increase in skin thickness was observed for both young and older mice (Figure 6D).

**Figure 7:**
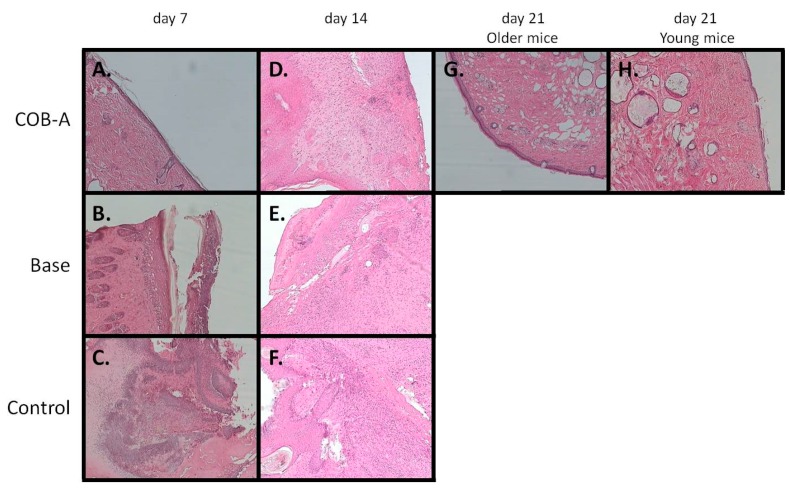
Representative histopathological images of mice skin on day 7 (**A**–**C**), day 14 (**D**–**F**) and day 21 (**G**–**H**) of treatment with 0.3% COB-A fraction (**A**, **D**, **G** and **H**) or the ointment base (**B** and **E**) and without treatment (**C** and **F**) (magnification 100× (**A**–**E** and **G**) and 200× (**F** and **H**)). Samples were stained with hematoxylin and eosin.

The evaluation of the histopathological sections of the differently treated mice groups revealed significant variation in the progress of the healing process (Figure 7). Indeed, the group treated with 2% COB extract showed the presence of only a few inflammatory cells on day 7, the healing process was initiated with the presence of fibroblasts being evident on day 14, while on day 21 still a few inflammatory elements were present in the epidermis and dermis. The group treated with 0.3% COB-A fraction did not show any signs of inflammation even on day 7, but instead evident was the initiation of the healing process which seemed to be completed by day 21. In contrast, the negative control group showed ulceration and the presence of crust with intense inflammatory infiltrations and inflammation in the dermis on day 7, which continued up to day 21 albeit to a lesser extent. Similar characteristics to the negative control group were observed for the group treated with the ointment base during the 21-day period.

Overall, the enriched in PUFAs COB-A fraction, which contained mainly EPA, exerted the best healing effect. The results were striking in both main healing criteria which include macroscopic evaluation regarding wound closure and histopathological evaluation that showed no inflammatory elements and almost normal skin re-establishment. Remarkably, it was the only group that showed fully healed mice by day 14.

Fatty acids in their phospholipidic forms are fundamental components of plasma membrane. In particular, the PUFAs present in plasma membrane, apart from their structural role, are also responsible for the modulation of cell–cell interactions and intracellular signal transduction [24]. Especially, n-3 and n-6 PUFAs are involved in the wound healing process, by participating in the biosynthesis of numerous lipoic mediators that have critical functions in inflammation, including vascular contraction, chemo-taxis, adhesion, and cellular activation [25,26].

Consequently, several investigations have been conducted for elucidating the competence of marine-derived fatty acids as wound healing agents [27]. Specifically, PUFAs have been investigated as wound healing accelerators via topical administration of codfish liver oil on murine skin wounds and were found to enhance the wound healing process [28]. Furthermore, lipids extracted from two different species of mollusks (the bivalve *Mytilus galloprovincialis* and the gastropod *Rapana venosa*) reduced the healing time of a skin burn wound inflicted on Wistar rats [29]. Besides, the potential of fatty acids isolated from the sea cucumber *Stichopus chloronotus* crude extract for wound healing has also been studied [30]. Conclusively, the effect of marine-derived n-3 PUFA on the production of proinflammatory cytokines in the wound serum and the time needed to achieve complete wound healing in healthy human skin has been evaluated using human participants and the potential of PUFAs in therapeutic applications in cutaneous wound healing was suggested via the increased proinflammatory cytokine production at wound sites [31].

Moreover, the presence of palmitoleic acid in the most active fraction tested in this study (COB-A) further enhances the wound healing process via its anti-inflammatory activity, which has been suggested to be responsible for healing, especially in the stages of granulation tissue formation and remodeling [32]. The moderate activity of the COB-B fraction could be attributed to the high content in oleic and linoleic acids, since previous studies have demonstrated that topical wound treatment with oleic and linoleic acids accelerates tissue repair mechanisms due to the ability of these fatty acids to modulate inflammation [33,34,35].

## 3. Materials and Methods

### 3.1. General Experimental Procedures

NMR spectra were recorded using Bruker DRX 400 (Bruker BioSpin GmbH, Rheinstetten, Germany). Chemical shifts are given on the *δ* (ppm) scale using TMS as internal standard. Gas chromatography-mass spectrometry (GC-MS) analyses of FAME were carried out using a Hewlett-Packard 6890 gas chromatograph (Hewlett-Packard Company, Wilmington, DE, USA) equipped with a HP-5MS fused silica capillary column (30 m × 0.25 mm, film thickness 0.25 μm) coupled to a Hewlett-Packard 5973 MS detector (Hewlett-Packard Company, Wilmington, DE, USA) operating in electron ionization mode at 70 eV. The GC-MS conditions for the analyses were the following: a split/splitless inlet at 280 °C was used at a split ratio 1:5; the carrier gas was He at a constant flow rate of 2 mL/min; the oven temperature was 60 °C at the time of the injection, raised to 240 °C at a rate of 3 °C/min, then raised to 300 °C at a rate of 10 °C/min, and finally maintained at 300 °C for 5 min; the MSD transfer line was set at 280 °C; the source temperature was set at 230 °C, while the quadrupole temperature was set at 150 °C. The identification of the chemical constituents was based on comparison of their Kováts indices, relative retention times and mass spectra with those obtained from authentic standards (Sigma Chemical Co., St. Louis, MO, USA, PhytoLab GmbH and Co., Germany, and in-house isolated and identified metabolites) and/or reported in the NIST/NBS and Wiley libraries. Vacuum column chromatography was performed with Kieselgel 60 (Merck, Darmstadt, Germany). Thin layer chromatography (TLC) was performed with Kieselgel 60 F_254_ aluminum plates (Merck, Darmstadt, Germany) and spots were visualized after spraying with 20% (v/v) H_2_SO_4_ in MeOH reagent and heating at 100 °C for 1 min.

### 3.2. Biological Material

Specimens of *C. oestroides* were removed from the buccal cavities of infected seabreams aquacultured at FORKYS fish farms in Chios island. A voucher specimen of the animal has been deposited at the animal collection of the Section of Pharmacognosy and Chemistry of Natural Products, Department of Pharmacy, National and Kapodistrian University of Athens (ATPH/MP0092).

### 3.3. Extraction and Fractionation

Freeze-dried *C. oestroides* (380 g) was exhaustively extracted with mixtures of CH_2_Cl_2_/MeOH at room temperature. The extract was evaporated *in vacuo* to afford an orange oily residue (COTOE, 90 g). Part of COTOE (50 g) was sequentially partitioned in water (500 mL) and organic solvents (500 mL, twice) of increasing polarity (cHex, CH_2_Cl_2_ and *n*-BuOH) to afford four extracts of different polarity, namely cHex (COA, 4.5 g), CH_2_Cl_2_ (COB, 20 g), *n*-BuOH (COC, 2.5 g) and H_2_O (COD, 23 g) extracts. Part of COB (5 g) was subjected to vacuum column chromatography on silica gel, using cHex with increasing amounts of EtOAc, to yield 12 fractions, which according to their TLC profiles were pooled together to afford 3 fractions (COB-A, COB-B, and COB-C) with distinct chemical profiles.

### 3.4. Derivatization of Fatty Acids

Fatty acid methyl esters (FAME) were prepared by transesterification. Briefly, 5% acetyl chloride in anhydrous MeOH (10 mL) were added to each sample (5 mg) and the mixture was heated at 80 °C for 1 h. After cooling, ddH_2_O (20 mL) was added. FAME were extracted after addition of cHex (20 mL). The FAME-containing organic layer was transferred to a glass vial, evaporated under nitrogen to a final volume of 2 mL, sealed with a Teflon-lined screw cap and stored at −20 °C until further analysis.

### 3.5. Animals and Study Design

All procedures performed were carried out in accordance with the guidelines established by the European Communities Council Directive (Directive 2010/63/EU of 22 September 2010). The experimental procedure was approved by the National Peripheral Veterinary Authority Animal Ethics Committee (Protocol Number: 717/11-02-2016) after the affirmative opinion of the Animal Protocols Evaluation Committee.

SKH-hr1 male and female mice (1–4 months of age) were used in this study. All mice originated from the breeding stock of the Small Animal Laboratory of the Department of Pharmacy (EL 25 BIO 07). The animal room was kept at 23 ± 1 °C and 25%–45% humidity and was illuminated by yellow fluorescent tubes in a 12 h light and dark cycle. The mice had unrestricted continuous access to standard chow diet (Nuevo SA-Farma-Efyra Industrial and Commercial SA, Greece) and fresh water.

The mice were divided into several groups (each consisting of 3–9 mice) receiving different treatments according to the experimental protocol. Specifically, for the first experiment the mice were divided into seven groups (n = 3–4): (1) mice treated with an ointment containing 1% COTOE extract, (2) mice treated with an ointment containing 1% COA extract, (3) mice treated with an ointment containing 1% COB extract, (4) mice treated with an ointment containing 1% COC extract, (5) mice treated with an ointment containing 1% COD extract, (6) mice treated with an ointment containing 1% COOE extract and (7) mice receiving no treatment (negative control). For the second experiment the mice were divided into six groups (n = 7): (1) mice treated with an ointment containing 1% COB extract, (2) mice treated with an ointment containing 1% COD extract, (3) mice treated with an ointment containing 1% COOE extract, (4) mice treated with the ointment base, (5) mice treated with Madecassol cream (*Centella asiatica* extract used as positive control) and (6) mice receiving no treatment (negative control). For the third experiment the mice were divided into eight groups (n = 4): (1) mice treated with an ointment containing 1% COB extract, (2) mice treated with an ointment containing 2% COB extract, (3) mice treated with an ointment containing 4% COB extract, (4) mice treated with an ointment containing 0.3% COB-A fraction, (5) mice treated with an ointment containing 0.3% COB-B fraction, (6) mice treated with an ointment containing 0.3% COB-C fraction, (7) mice treated with the ointment base and (8) mice receiving no treatment (negative control). For the fourth and final experiment the mice were divided into four groups (n = 9): (1) mice treated with an ointment containing 2% COB extract, (2) mice treated with an ointment containing 0.3% COB-A fraction, (3) mice treated with the ointment base and (4) mice receiving no treatment (negative control).

The ointment base consisted of 64% petrolatum, 9.5% squalene, 9.5% olive oil, 4.7% calendula oil, 4.7% beeswax, 4.7% propylene glycol, 2% Vitamin E, and 0.9% α-lipoic acid.

In all experiments, the wound was cleaned daily with diluted soap 50% in saline, rinsed with saline solution and either treated topically with the extract preparations or left without treatment (negative control).

### 3.6. Infliction of Wounds

The animals were initially anaesthetized using ketamine (100 mg/mL) and xylazine (20 mg/mL) at a 3:1 v/v ratio and subsequently a 1 cm^2^ piece of skin was surgically removed from the dorsal region of each mouse. After skin excision, the wound was cleaned initially with diluted soap 50% in saline, rinsed with saline solution and treated topically with the extract preparations (10 mg for the wound and 10 mg for the area around the wound). The mice were maintained in individual cages under a warming lamp and were monitored until fully recovered from the anesthesia.

### 3.7. Clinical Evaluation, Photo-Documentation, and Histopathological Analysis

The clinical condition of mice (e.g., total wound area, progress of healing process, skin inflammation intensity and mobility) was recorded daily. The animals were weighted periodically. Skin images for photo-documentation were acquired using a Nikon D5100 digital camera (Nikon, Tokyo, Japan) equipped with an AF-S Micro Nikkor 60 mm f/2.8 G ED lens (Nikon, Tokyo, Japan), which was at a fixed distance of 30 cm perpendicular to the subject. The photographs were digitized, and the wound area was measured using Adobe Photoshop C5. Wound closure was defined as a reduction in the wound area and the results were expressed as a percentage (%) of the original wound area.

At the end of the experiment, when total wound closure could be observed for at least one group of treated mice, the mice were sacrificed and skin tissue was excised. Skin sections of specimens from all groups were performed using a paraffin microtome (Shandon Finesse, Thermo Fisher Scientific, Cheshire, UK) and stained with hematoxylin and eosin stain kit (Atom Scientific, Cheshire, UK). Parameters such as inflammation, hyperkeratosis, parakeratosis and skin structure, were estimated.

### 3.8. Evaluation of Skin Parameters

Skin parameters, including hydration, TEWL, elasticity and skin thickness, were evaluated with non-invasive biophysical methods. Hydration was measured through changes in the dielectric constant using a Corneometer CM 820 (Courage + Khazaka electronic GmbH, Köln, Germany). The corresponding probe was applied and the indications were recorded in arbitrary units. The water barrier function of skin (TEWL) was evaluated by measuring the density gradient of the water evaporation from the skin using a Tewameter TM 210 (Courage + Khazaka electronic GmbH, Köln, Germany), with the estimation being based on the mean value of the flux density of water (in g/m^2^/h) which was obtained 1 min after the beginning of the measurement. Skin thickness, evaluated on skin fold treated areas, was measured with a digital micrometer caliper Powerfix Profi (Milomex Ltd, Bedfordshire, UK). Elasticity was measured using a Cutometer MPA 580 (Courage + Khazaka electronic GmbH, Köln, Germany), after probe pumping and release of the treated skin, by calculating the R2 parameter (gross elasticity equaling resistance versus ability of returning, where 1 corresponds to 100% elasticity). Before each measurement, the treated area was cleaned by wiping the skin surface with sterile gauze.

### 3.9. Statistical Analysis

The results were expressed as mean values ± SD. Statistical differences were estimated using analysis of variance (ANOVA) single factor test and were considered significant when *p* < 0.05.

## 4. Conclusions

A series of different polarity (organic and aqueous) extracts of the marine isopod *C. oestroides* was prepared and evaluated for their in vivo wound healing activity after topical administration in the form of ointments on wounds inflicted on SKH-hr1 hairless mice. Wound healing was evaluated primarily by daily clinical assessment and photo-documentation, as well as histopathological analysis and measurement of biophysical skin parameters (TEWL, hydration, elasticity and skin thickness). On the basis of the results of the preliminary evaluation of the different extracts, the CH_2_Cl_2_ (COB) one that was considered as the most promising (exhibiting very few inflammatory elements in the histopathological analysis), was selected for further investigation. Following chromatographic fractionation, the obtained fractions were further evaluated for their wound healing activity, while their chemical profiles were analyzed. The highest levels of activity were exerted by treatment of the wounds with a fraction rich in EPA, myristic and palmitoleic acids. Topical application of the bioactive fraction on the wounds of mice resulted in complete wound closure and skin of an almost normal architecture devoid of inflammatory elements.

## Supplementary Materials

The following are available online at https://www.mdpi.com/1660-3397/18/4/219/s1, Figures S1–S7: Various visual representations of results discussed in the text.
