# Supplementary material for: In Vivo Evaluation of the Wound Healing Activity of Extracts and Bioactive Constituents of the Marine Isopod Ceratothoa oestroides
Source: Mar Drugs. 2020 Apr 18;18(4):219. doi: 10.3390/md18040219 (PMC7230750; doi:10.3390/md18040219)
Supplement: Supplementary file 1 [file marinedrugs-18-00219-s001.pdf]

## Supplementary Materials for

# *In vivo* Evaluation of the Wound Healing Activity of Extracts and Bioactive Constituents of the Marine Isopod *Ceratothoa oestroides*

**Evgenia Sofrona [1,2], Leto-Aikaterini Tziveleka [1], Maria Harizani [1], Panagiota Koroli [2], Ioannis Sfiniadakis [3], Vassilios Roussis [1], Michail Rallis [2,*] and Efstathia Ioannou [1,*]**

[1] Section of Pharmacognosy and Chemistry of Natural Products, Department of Pharmacy, School of Health Sciences, National and Kapodistrian University of Athens, Athens 15771, Greece; evgenia.sofrona@gmail.com (E.S.); ltziveleka@pharm.uoa.gr (L.A.T.); mariachariz@pharm.uoa.gr (M.H.); roussis@pharm.uoa.gr (V.R.)

[2] Section of Pharmaceutical Technology, Department of Pharmacy, School of Health Sciences, National and Kapodistrian University of Athens, Athens 15784, Greece; giotakor17@hotmail.com (P.K.)

[3] Athens Naval Hospital, Pathologoanatomic Laboratory, Athens 11521, Greece; jsfiniadakis@yahoo.gr (I.S.)

* Correspondence: rallis@pharm.uoa.gr (M.R.); eioannou@pharm.uoa.gr (E.I.); Tel.: +30-210-7274699 (M.R.); +30-210-7274913 (E.I.).

# Table of Contents

**Figure S1.** TEWL values for the various mice groups receiving different treatments (no treatment, control; 1% olive oil extract, COOE; 1% total organic extract, COTOE; 1% cHex extract, COA; 1% $CH_2Cl_2$ extract, COB; 1% *n*-BuOH extract, COC; 1% $H_2O$ extract, COD) on day 18 of the experiment. Values are presented as the mean±SD (n=3-4 mice per group). One-way ANOVA followed by Tukey's test was applied for comparison between the extract-treated mice groups and the negative control (*$p$<0.05 vs control). S3

**Figure S2.** (**A**) TEWL values for male and female mice groups receiving different treatments (ointment base, BASE; Madecassol; 1% olive oil extract, COOE; 1% $CH_2Cl_2$ extract, COB; 1% $H_2O$ extract, COD) on day 1 and day 25 of the experiment. (**B**) Hydration values for male and female mice groups receiving different treatments on day 1 and day 25 of the experiment. (**C**) Skin elasticity values for male and female mice groups receiving different treatments on day 1 and day 25 of the experiment. (**D**) Skin thickness values (mm) for male and female mice groups receiving different treatments on day 1 and day 25 of the experiment. Values are presented as the mean±SD (n=7 animals per group). One-way ANOVA followed by Tukey's test was applied for comparison between the treated mice and the control (*$p$<0.05 vs control). S3

**Figure S3.** Representative histopathological images of male and female mice skin after treatment with the ointment base (**A** and **B**, respectively), Madecassol (**C** and **D**, respectively), 1% COB extract (**E** and **F**, respectively) or 1% COD extract (**G** and **H**, respectively) (magnification 100× (**C**-**G**), 200× (**A**, **B**) and 400× (**H**)). Samples were stained with hematoxylin and eosin. S4

**Figure S4.** $^1$H NMR spectrum of fraction COB-A in $CDCl_3$. S5

**Figure S5.** $^1$H NMR spectrum of fraction COB-B in $CDCl_3$. S5

**Figure S6.** $^1$H NMR spectrum of fraction COB-C in $CDCl_3$. S6

**Figure S7.** (**A**) TEWL values for the various mice groups receiving different treatments (no treatment, control; ointment base, BASE; 1%, 2%, and 4% $CH_2Cl_2$ extract, COB1%; COB2% and COB4%, respectively; 0.3% COB-A fraction, COB-A; 0.3% COB-B fraction; COB-B; 0.3% COB-C fraction, COB-C) on day 1 and day 24 of the experiment. (**B**) Hydration values for the various mice groups on day 1 and day 24 of the experiment. Values are presented as the mean±SD (n=4 mice per group). One-way ANOVA followed by Tukey's test was applied for comparison between the treated mice and the control (*$p$<0.05 vs control). S6

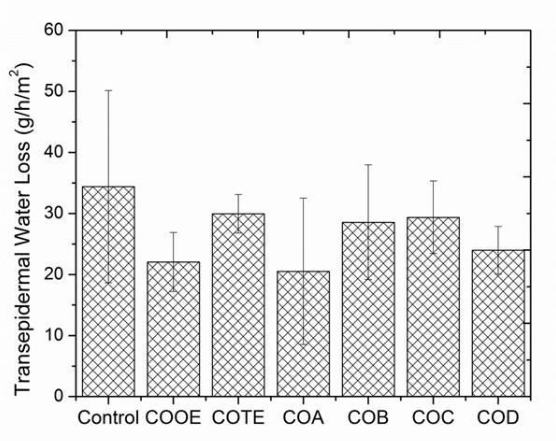


**Figure S1.** TEWL values for the various mice groups receiving different treatments (no treatment, control; 1% olive oil extract, COOE; 1% total organic extract, COTOE; 1% cHex extract, COA; 1% $CH_2Cl_2$ extract, COB; 1% *n*-BuOH extract, COC; 1% $H_2O$ extract, COD) on day 18 of the experiment. Values are presented as the mean±SD (n=3-4 mice per group). One-way ANOVA followed by Tukey's test was applied for comparison between the extract-treated mice groups and the negative control (*$p$<0.05 vs control).

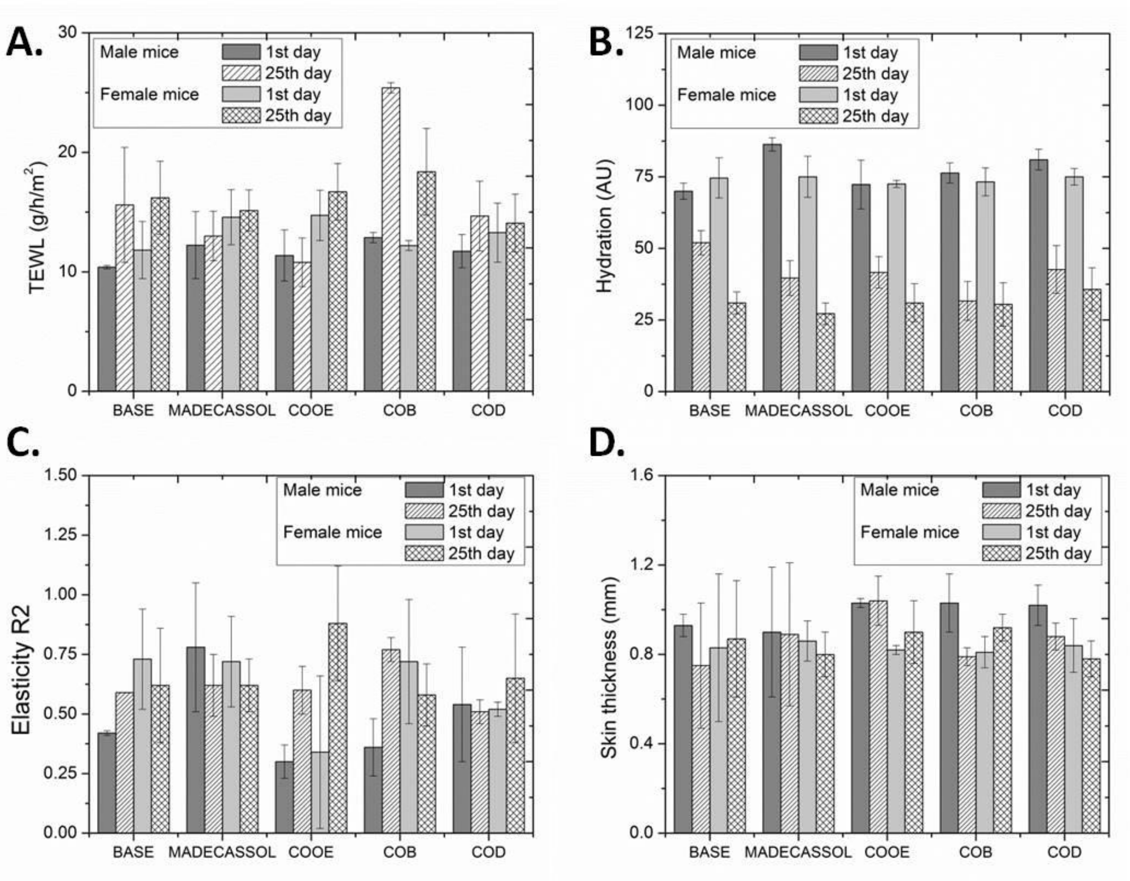


**Figure S2.** (**A**) TEWL values for male and female mice groups receiving different treatments (ointment base, BASE; Madecassol; 1% olive oil extract, COOE; 1% $CH_2Cl_2$ extract, COB; 1% $H_2O$ extract, COD) on day 1 and day 25 of the experiment. (**B**) Hydration values for male and female mice groups receiving different treatments on day 1 and day 25 of the experiment. (**C**) Skin elasticity values for male and female mice groups receiving different treatments on day 1 and day 25 of the experiment. (**D**) Skin thickness values (mm) for male and female mice groups receiving different treatments on day 1 and day 25 of the experiment. Values are presented as the mean±SD (n=7 animals per group). One-way ANOVA followed by Tukey's test was applied for comparison between the treated mice and the control (*$p$<0.05 vs control).

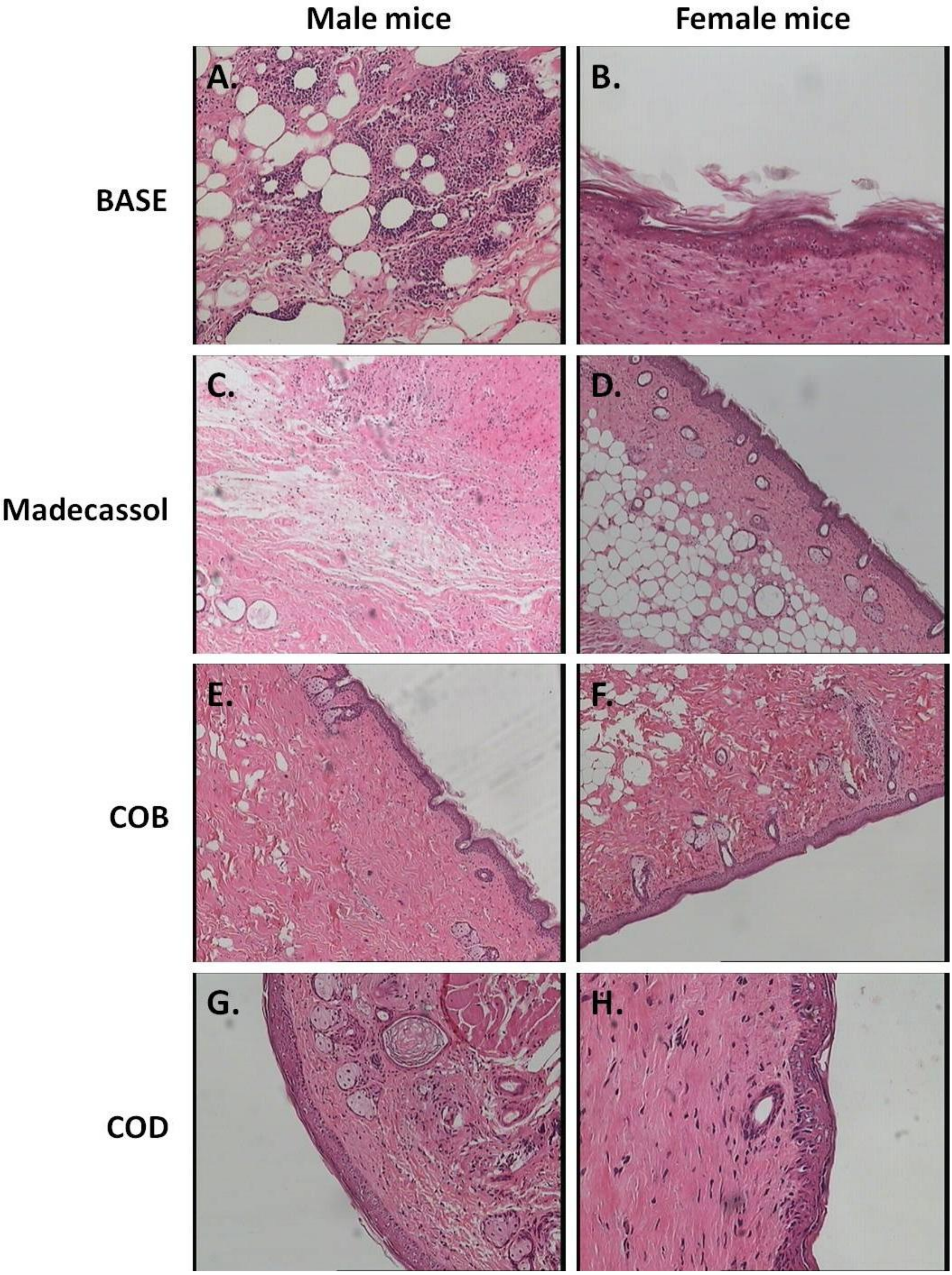


**Figure S3.** Representative histopathological images of male and female mice skin after treatment with the ointment base (**A** and **B**, respectively), Madecassol (**C** and **D**, respectively), 1% COB extract (**E** and **F**, respectively) or 1% COD extract (**G** and **H**, respectively) (magnification 100× (**C**-**G**), 200× (**A**, **B**) and 400× (**H**)). Samples were stained with hematoxylin and eosin.

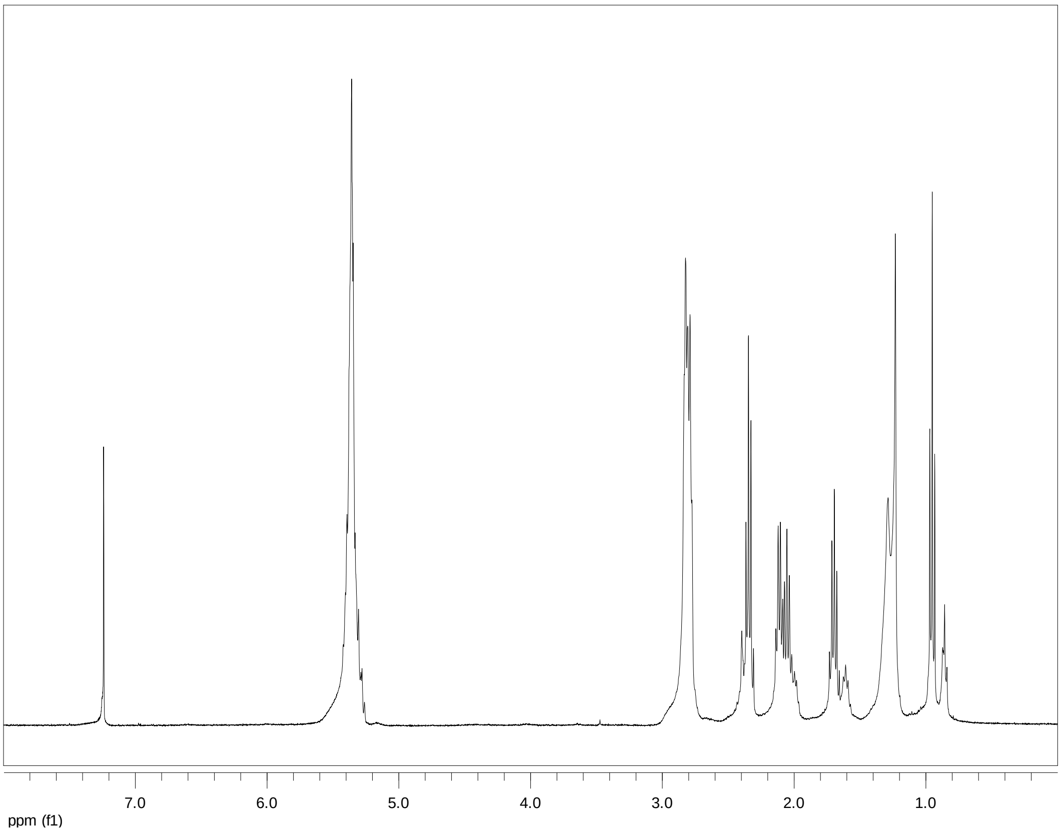


**Figure S4.** $^1$H NMR spectrum of fraction COB-A in $CDCl_3$.

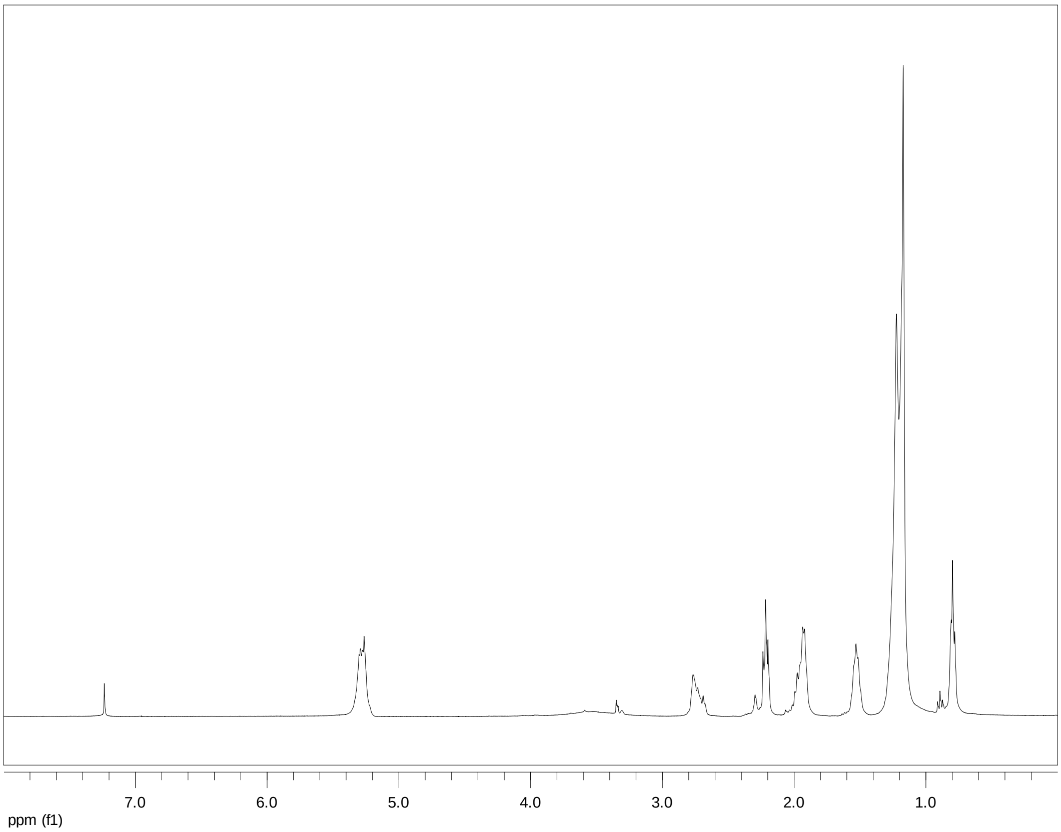


**Figure S5.** $^1$H NMR spectrum of fraction COB-B in $CDCl_3$.

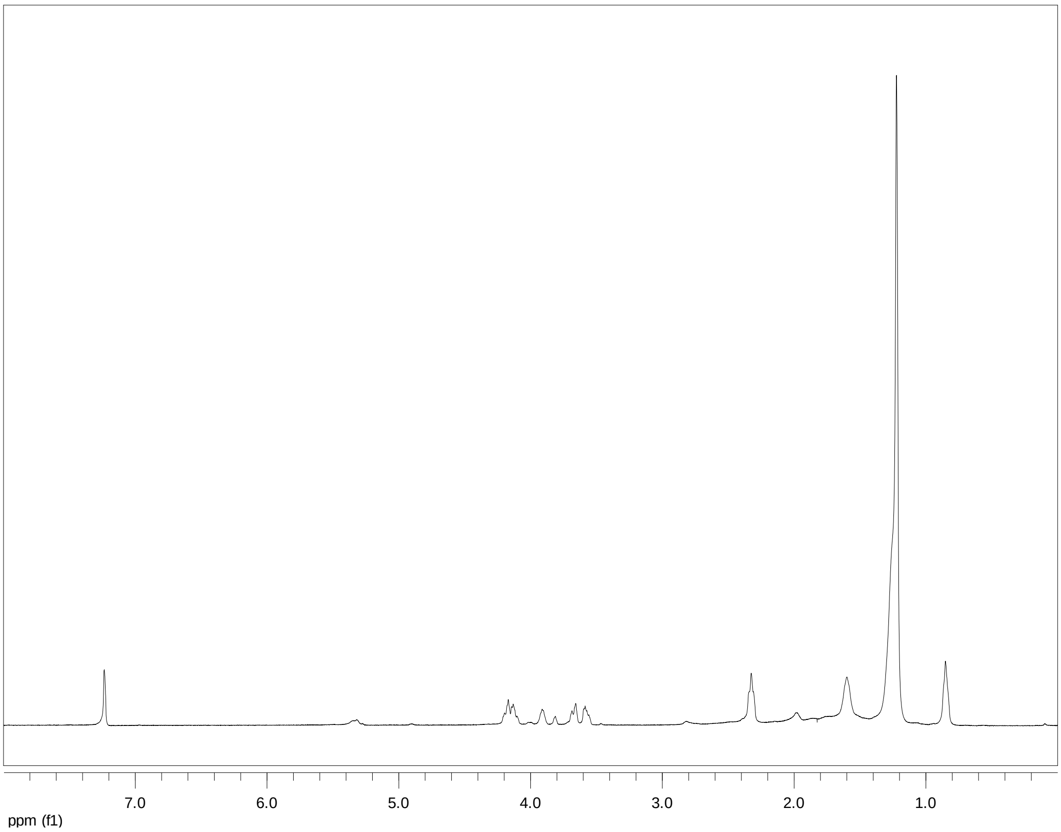


**Figure S6.** $^1$H NMR spectrum of fraction COB-C in $CDCl_3$.

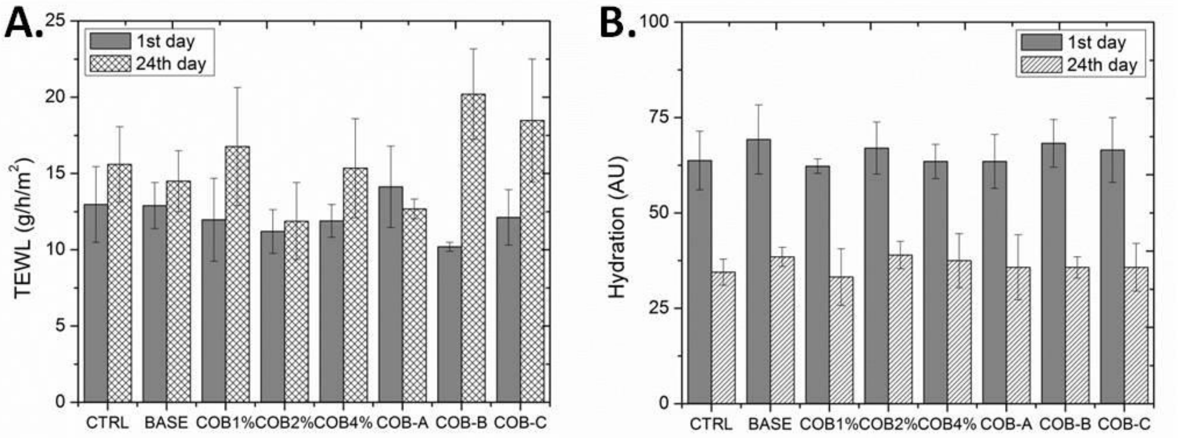


**Figure S7.** (**A**) TEWL values for the various mice groups receiving different treatments (no treatment, control; ointment base, BASE; 1%, 2%, and 4% $CH_2Cl_2$ extract, COB1%; COB2% and COB4%, respectively; 0.3% COB-A fraction, COB-A; 0.3% COB-B fraction; COB-B; 0.3% COB-C fraction, COB-C) on day 1 and day 24 of the experiment. (**B**) Hydration values for the various mice groups on day 1 and day 24 of the experiment. Values are presented as the mean±SD (n=4 mice per group). One-way ANOVA followed by Tukey's test was applied for comparison between the treated mice and the control (**p*<0.05 vs control).
